# A novel technique of serial biopsy in mouse brain tumour models

**DOI:** 10.1371/journal.pone.0175169

**Published:** 2017-04-10

**Authors:** Sasha Rogers, Hilary Hii, Joel Huang, Mathew Ancliffe, Nick G. Gottardo, Peter Dallas, Sharon Lee, Raelene Endersby

**Affiliations:** 1 Telethon Kids Institute, Perth, WA, Australia; 2 Centre for Child Health Research, University of Western Australia, Perth, WA, Australia; 3 Department of Neurosurgery, Princess Margaret Hospital, Perth, WA, Australia; 4 Department of Oncology, Princess Margaret Hospital, Perth, WA, Australia; Universidad de Navarra, SPAIN

## Abstract

Biopsy is often used to investigate brain tumour-specific abnormalities so that treatments can be appropriately tailored. Dacomitinib (PF-00299804) is a tyrosine kinase inhibitor (TKI), which is predicted to only be effective in cancers where the targets of this drug (EGFR, ERBB2, ERBB4) are abnormally active. Here we describe a method by which serial biopsy can be used to validate response to dacomitinib treatment *in vivo* using a mouse glioblastoma model. In order to determine the feasibility of conducting serial brain biopsies in mouse models with minimal morbidity, and if successful, investigate whether this can facilitate evaluation of chemotherapeutic response, an orthotopic model of glioblastoma was used. Immunodeficient mice received cortical implants of the human glioblastoma cell line, U87MG, modified to express the constitutively-active EGFR mutant, EGFRvIII, GFP and luciferase. Tumour growth was monitored using bioluminescence imaging. Upon attainment of a moderate tumour size, free-hand biopsy was performed on a subgroup of animals. Animal monitoring using a neurological severity score (NSS) showed that all mice survived the procedure with minimal perioperative morbidity and recovered to similar levels as controls over a period of five days. The technique was used to evaluate dacomitinib-mediated inhibition of EGFRvIII two hours after drug administration. We show that serial tissue samples can be obtained, that the samples retain histological features of the tumour, and are of sufficient quality to determine response to treatment. This approach represents a significant advance in murine brain surgery that may be applicable to other brain tumour models. Importantly, the methodology has the potential to accelerate the preclinical *in vivo* drug screening process.

## Introduction

Brain biopsy is a commonly used clinical procedure that facilitates extraction of brain tissue for histological analysis [1], and the subsequent initiation of appropriate treatment based on accurate morphological diagnosis [2]. Recent advances in genomic profiling techniques have enabled rapid identification of mutations and signalling pathway deregulation leading to a paradigm shift in the way that tumours are defined. Traditional histological analysis of tumours is being superseded by molecular profiling [3],[4], providing a platform for the development of “targeted therapies” [5] and individually tailored treatment protocols that optimise patient outcomes while minimizing side effects.

Over recent years, mouse models have been indispensable for modelling rare human tumours. Sophisticated techniques have been developed to generate tumours driven by specific aberrant pathways known to be associated with tumour subtypes [3]. Translating biopsy techniques to the preclinical setting has the potential to provide evidence of response to treatment with novel chemotherapeutic agents.

Immunohistological analysis remains the gold standard to identify deregulated signalling pathways and changes in protein expression that may occur within a tumour in response to treatment. However, needle biopsy is largely impractical in mouse models due to the size of traditional biopsy tools relative to murine tissues, and *ex vivo* histological analysis and live animal imaging are more commonly used in this context. Although the sophisticated tools and techniques that have been developed to assess the deregulation of specific genes/pathways are useful for longitudinal *in vivo* studies of brain diseases, these methods are generally limited to inducible models designed to express specific reporters [6,7]. The traditional alternative involves standard histological techniques that involve tissue harvesting; a process which is not only time consuming and involves a large number of animals, but also relies on assumptions of similarity between animals for histological comparison.

A major goal of preclinical research is to determine if novel oncological therapies are effective in different cancer models. Receptor tyrosine kinase (RTK) inhibitors are frequently being developed for cancer therapy. In order to determine if these agents are efficacious, it is imperative to first ensure that the drug is able to inhibit its intended target *in vivo*. U87MG is a widely used model of human GBM, and its rapid growth in mouse brain is ideally suited for preclinical research. The model we developed utilised the U87MG cell line which was modified to express the EGFRvIII mutation that is found in up to 50% of human GBM. Not only is this variant the most common EGFR mutant, its enhanced tumourigenicity combined with the lack of EGFRvIII expression in normal tissue makes it an ideal candidate to assess targeted therapy [6]. By forced expression of active EGFR in the tumour, we sought to determine if inhibition of the receptor could be detected in biopsied tissue. To this end, we used the pan-ERBB inhibitor, dacomitinib (PF-00299804). This drug is a second-generation irreversibly-binding inhibitor, which is reported to inhibit wild-type ERBB receptors as well as mutated forms of EGFR resistant to the first generation reversible inhibitors, gefitinib or erlotinib [7].

We describe a unique method for performing serial free-hand biopsies at various time points during treatment in a mouse model of glioblastoma (GBM). We propose that conducting serial biopsies in animal models will help streamline the process of preclinical testing of novel drug treatments, with the added benefit of reducing the number of animals required for the screening process.

## Materials and methods

### 2.1 Cell culture

Human U87MG GBM cells acquired from the ATCC were cultured in DMEM containing antibiotics (penicillin/streptomycin), glutamax and 10% fetal bovine serum. The cells were transduced with retrovirus to enable expression of a constitutively active form of the EGF receptor (EGFRvIII), green fluorescent protein (GFP) and a puromycin acetyltransferase/luciferase fusion protien (pacLuc2), using the retroviral expression constructs MSCV-EGFRvIII-ires-GFP and MSCV-ires-pacLuc2, respectively. The constructs were kindly provided by Drs Suzanne Baker and Richard Williams of St Jude Children’s Research Hospital (Memphis, USA). In preparation for intracranial implantation, cells were trypsinised, washed, and resuspended in matrigel to a final concentration of 50,000 cells per microliter.

### 2.2 Intracranial implantation

All animal research was conducted according to protocols approved by the Telethon Kids Institute Animal Ethics Committee based on the NHMRC “Australian code for the care and use of animals for scientific purposes” guidelines. Balb/c nude mice were purchased from the Animal Resources Centre (Murdoch, Western Australia) and housed in sterile individually ventilated cages with access to food and water *ad libitum*. All mice were given subcutaneous buprenorphine injections (0.1mg/kg) as pre-emptive analgesia. Mice under deep anesthesia after intraperitoneal injection (i.p) with ketamine/medetomedine cocktail (100mg/kg and 10mg/kg, respectively), were ear notched and prepared for surgery with alcohol and iodine swabs to sterilise the scalp. Mice were placed in a stereotactic frame (Kopf) and a small linear incision was made over the right frontal region. The pericranium was gently removed and a small burr hole was made using a dental drill (Osada) 1mm behind the Bregmatic suture and 3mm lateral to the midline. The dura was exposed and opened, and the brain surface washed with artificial cerebrospinal fluid (aCSF). The previously resuspended cells were drawn up in to a sterile Hamilton Syringe bearing a 26G unbevelled needle for implantation. The syringe was then placed in the z-axis of the stereotactic apparatus and used to accurately deliver cells at a depth of 1.5mm into the cortex using specific coordinates defined for the mouse brain in the Mouse Brain Atlas [8]. After implantation of 1μL of cell suspension the wound was sealed with cyanoacrylate and the mice were given subcutaneous carprofen (5mg/kg) for analgesia, and atipamezole (0.5mg/kg) to reverse the effects of medetomidine. To alleviate pain, mice were given ibuprofen (115 μg/mL) in drinking water for four days as a post-operative analgesic.

### 2.3 Postoperative imaging

After intracranial implant, tumour growth was assessed once-weekly using bioluminescence imaging starting seven days post-surgery. Mice were injected with 150mg/kg i.p. D-Luciferin (the substrate of the firefly luciferase enzyme, purchased from Caliper Life Sciences), anaesthetised with 2% isoflurane in oxygen and images were captured every minute over a 20 minute interval using an IVIS Spectrum *in vivo* bioimaging system (Caliper). The intensity (photons/sec) of light in captured images was quantified using the LivingImage 4.3 software.

### 2.4 Drug treatment

Dacomitinib (PF-00299804) was kindly provided by Pfizer (New York City, NY, USA). The drug was administered orally at 30mg/kg in 50mM sodium lactate buffer (pH 4).

### 2.5 Open biopsy technique

Once tumour bioluminescence reached a predetermined level (approximately 10^7^ photons/sec), mice were given pre-emptive analgesia (subcutaneous (s.c) buprenorphine; 0.01mg/kg) 30 minutes prior to surgery. The animals were prepared for surgery with alcohol and iodine swabs to sterilise the scalp. The eyes were protected from drying out with petrolatum ophthalmic ointment. Once anaesthesia was at an appropriate level, mice were placed in a stereotactic frame. The old incision from stereotactic implantation was re-opened and slightly extended as necessary to expose the previous burr hole. Using a dental drill, the burr hole was expanded slightly to gain access to the implanted cortex and tumour. A digital camera (Leica) and a light source and emission filter to detect GFP, were used to distinguish tumour from normal cortex. Under low magnification (Leica) a section of tissue from within the region of GFP fluorescence was selected for resection. Using McPherson-Vannas microdissection scissors (Roboz) a small incision was made through the pia mata and extended around in a small square. The incision was deepened slightly and undercut to release it from the underlying white matter. The tissue sample was gently teased out using forceps, and scissors were used to dissect it completely from the surrounding tissue. Tissue was fixed by immersion in to 4% paraformaldehyde (PFA) in PBS for 15 minutes. The samples were cryoprotected by immersion in 25% sucrose in PBS overnight prior to freezing in optimal cutting medium (OCT, Sakura). Any bleeding encountered during the procedure stopped by gentle irrigation with artificial CSF, followed by application of gentle pressure and oxidized regenerated cellulose haemostatic agent (Surgicell, Fibrillar (Ethicon)). Once haemostasis was achieved, any remaining haemostatic material was meticulously removed as it blocked the light produced by luciferase, and interfered with bioluminescence imaging. Following the procedure, the wound edges were opposed and sealed with cyanoacrylate glue. To alleviate pain, mice were given ibuprofen (115 μg/mL) in drinking water for four days as a post-operative analgesic.

### 2.6 Postoperative neurological injury severity assessment

After biopsy, each mouse was evaluated daily using a ten-point neurological severity score (NSS), slightly modified from the system described by Beni-Adani [9]. A description of the NSS criteria is presented in Table 1. For each failed task the mouse receives 1 point. The Maximum NSS equals 10 (failed all tasks). NSS was assessed for all animals in control and biopsy groups by two independent observers and compared with tumour-bearing animals that were not biopsied to ensure any changes in neurology were biopsy-related and not caused by tumour progression. Individual mice were placed in a purpose built obstacle course and assessed according to the NSS criteria on a daily basis. The assessments were carried out by two independent observers (SR and JH–a veterinarian not directly involved with the biopsy process). The scores were recorded for animals undergoing first biopsy until baseline NSS was reached, and then again after a second biopsy. A control group included tumour-bearing animals that did not undergo the biopsy procedure so as to address any neurological deterioration related to tumour progression.

**Table 1:** Criteria for neurological injury assessment based on a neurological severity score (NSS).

Task	NSS points
Presence of mono or hemiparesis	1
Inability to walk on 3cm wide beam	1
Inability to walk on 2cm wide beam	1
Inability to walk on 1cm wide beam	1
Inability to balance on 0.5cm wide beam	1
Inability to balance on 0.5cm diameter round stick	1
Failure to exit 30cm circle within 2 minutes	1
Inability to walk straight	1
Loss of startle reflex	1
Loss of seeking behaviour	1
**Maximum**	**10**

### 2.7 Animal euthanasia and tissue collection

This study was carried out in strict accordance with the recommendations in the “Guide for the Australian Code of Practice for the care and Use of Animals for Scientific Purposes”. The protocol was approved by the Animal ethics committee of the Telethon Kids Institute Western Australia (Permit Number: AEC#:241).

Animals were monitored daily for signs of tumour-related morbidity including head doming, lethargy, ataxia, and weight loss. Upon the onset of symptoms mice were euthanized using an anaesthetic overdose of avertin (500mg/kg i.p 2-2-2 tribromoethanol in phosphate buffered saline [PBS]). Once unresponsive, mice were transcardially perfused with 10mL of PBS. Brains were harvested and immersion fixed in 4% PFA in PBS at 4°C overnight. The brains were then divided coronally across the tumour site. One half was cryoprotected in 25% sucrose in PBS overnight, and the other half was processed and embedded into paraffin.

### 2.8 Immunohistochemistry

Biopsy samples were transferred to Superfrost(Menzel-Glaser) glass slides (Thermo-fisher) after cryo-sectioning at 10μm. Antigen retrieval was performed after three washes in PBS solution to remove excess OCT. Slides were incubated for five mins at room temperature after covering sections in Antigen retrieval was performed using 1% sodium dodecyl sulphate (SDS) in PBS as previously described [10]. Residual SDS was removed by a further three washes in PBS before transferring to Tris-buffered saline (TBS) for preparation of initial antigen blocking in TBS containing 10% normal goat serum, 0.01% Thimerosal and 0.01% Tween-20. Slides were incubated for 1 hour at room temperature before primary antibody incubation overnight (4^°^C) for phospho-EGFR (Y1068) (Cell Signaling, 2234) diluted in antibody diluent (TBS containing 2% normal goat serum, 0.01% thimerosal and 0.01% Tween-20) at 1:200. After three washes in 1xTBS/0.01% Tween-20, antibody binding was detected using DAB according to the manufacturer’s instructions (Vector Laboratories). Slides were rinsed in distilled water and counterstained for 5 minutes using Gill’s Haematoxylin, dipped in 2% acetic acid, dipped in distilled water and bluing agent for 45 Seconds before final dehydration in 30 second steps of 20%,50%,70%,95%, 100% ethanol, and finally xylene. Sections were mounted in Permount (Fisher).

## Results

### 3.1 Biopsy of mouse brain is associated with minimal neurological morbidity

**Fig 1:**
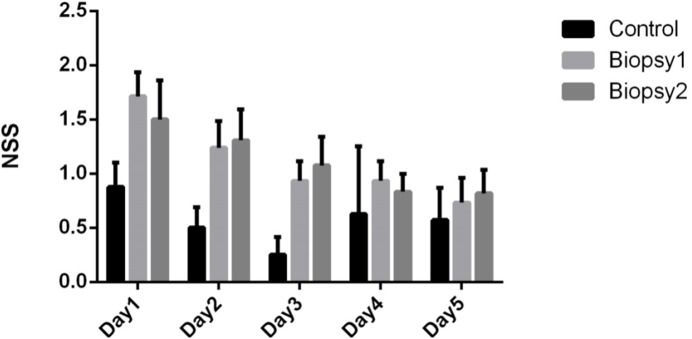
Biopsy-related neurological morbidity is minimal and reversible. Mean neurological severity score (NSS, error bars indicate standard deviation) was assessed for all animals in control (tumour-bearing, no biopsy) and biopsy groups. Controls (n = 8) were assessed for five days. “Biopsy 1” animals (n = 22) were assessed for 5 days after one biopsy. “Biopsy 2” mice (n = 14) having recovered after biopsy one, were biopsied a second time and assessed for another 5 days. The biopsy procedure was associated with minimal morbidity and mice recovered to scores equal to controls by day 5 (NSS = 0.73 vs 0.57, p = 0.68). After a second biopsy there was minimal initial neurological injury (NSS = 1.5) on day 1 after biopsy, which returned to baseline level by day 5 (NSS = 0.81 vs 0.57, p = 0.51).

To determine if biopsy of mouse brain tumours is a viable procedure, we induced tumours in mouse brain by intracranial implantation of human GBM cells that expressed GFP and luciferase. A biopsy technique was then developed and performed to determine if histological information could be gained from the extracted tissue, and to ensure animals were able to tolerate the procedure. The process of brain biopsy involves a significant risk of morbidity and mortality particularly in eloquent brain regions. To ensure animal welfare was maintained we incorporated a means to measure neurological injury related to the procedure, using a validated NSS previously established for animal models of head injury [9]. This 10 point scoring system evaluated seeking behaviour, balance, and ability to walk on various platforms of reducing width. Animals were assessed daily after surgery in a purpose built course, to identify any effects of neurological injury. In order to ensure the scoring was unbiased, assessments were conducted by the main author (SR) and a veterinarian (JH). Despite the relatively large tissue samples obtained (approximately 1mm^3^), very little morbidity resulted from the procedure on day 1 after first biopsy (Mean NSS = 1.7, range 0–3). So as not to confound the scoring with any potential neurological deterioration related to tumour progression, a group of control mice was included (n = 8) that underwent implantation at the same time as the first biopsy group. This cohort underwent daily assessment concurrently with the biopsy group for five days after first biopsy (Fig 1, *control*). Notably, neurological morbidity returned to baseline by day 5 after biopsy. The mice in the operative group then underwent drug treatment (discussed further below) and a subsequent second biopsy was performed at the original site. Again, minimal neurological morbidity was noted (mean NSS = 1.5, range 0–4), which returned to baseline after five days. No statistically significant differences in NSS between each of the groups were detected.

The biopsy procedure itself did not result in animal mortality. All mice that required euthanasia due to high tumour burden, which correlated with high bioluminescence readings. Failure to recover from anaesthesia was observed in one instance, which is a rare but not uncommon occurrence as maintenance of mice in a uniform plane of anaesthesia is challenging. These data show that multiple biopsies of mouse brain tumours are achievable, and neurological sequelae as a consequence of the procedure are transient and reversible.

### 3.2 Serial biopsy provides adequate tissue samples for histological analysis

**Fig 2:**
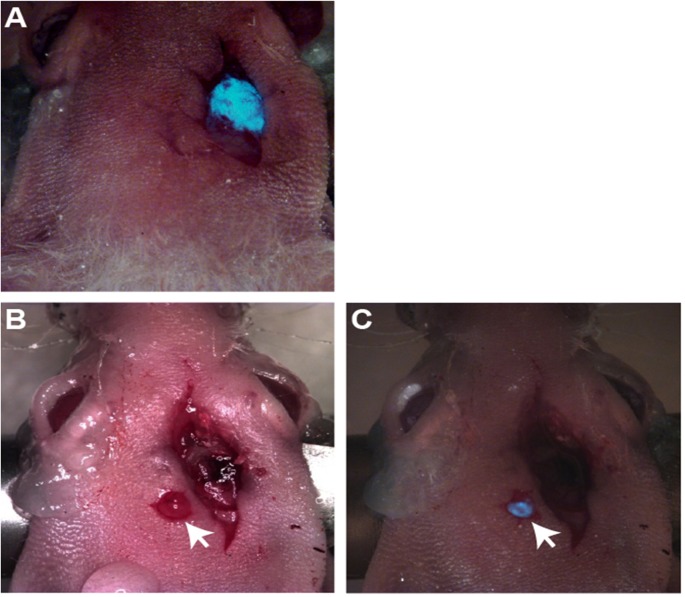
Micro-dissection can successfully remove a tumour biopsy sample from the murine cerebral cortex. Intraoperative images showing a fluorescent tumour *in situ* expressing GFP (A) and after removal, where the biopsied tissue is marked with a white arrow (B and C).

We wanted to determine if biopsied tissue was histologically informative in a preclinical setting. The biopsy procedure was significantly aided by using an orthotopic transplant model, where the cells had been modified to express a fluorescent marker, in this case GFP. This enabled accurate location of the tumour *in situ* (Fig 2A). After removal of the tumour sample, detection of GFP was again employed to confirm that the tissue sample consisted mainly of tumour cells, with minimal or no normal brain tissue contamination (Fig 2B & 2C).

**Fig 3:**
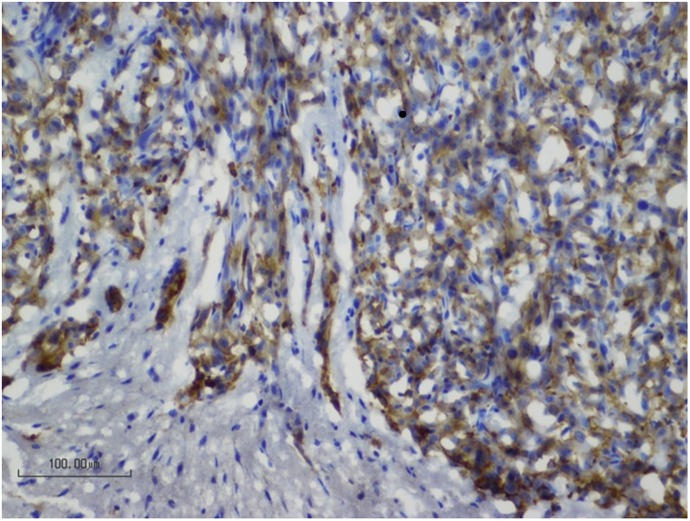
Histological integrity is maintained in biopsy samples. A representative image of a paraffin section from a biopsy sample. Immunohistochemical staining for phosphorylated EGFR (brown) illustrates a clear demarcation between the EGFRvIII expressing tumour cells and normal brain. Sections were counterstained with haematoxylin (blue). Scale bar represents 100μm.

All biopsy samples were fixed and snap frozen in OCT prior to sectioning and histochemical staining. The main difficulties encountered were related to the processing of the very small tissue samples. Cryosections were preferred over paraffin embedded tissue because the biopsy samples were so small that paraffin processing was impractical. The traditional antigen retrieval process of boiling sections prior to immunohistochemistry initially led to loss of the samples from slides. Instead, incubating slides in 1% SDS at room temperature for 5 mins [11] provided excellent antigen retrieval, while maintaining the integrity of the delicate sections (Fig 3). Despite the small size and minor artefacts from processing (such as ice crystals) of the extracted tissue, adequate tissue samples were reliably obtained from all animals.

### 3.3 Tissue obtained by biopsy can be used to determine effects of treatment

Previous work determined that inhibition of EGFRvIII phosphorylation by dacomitinib could be detected in whole tumour-bearing mouse brain at two hours post-treatment (S1 Fig).

To determine if inhibition of EGFRvIII phosphorylation could be detected in biopsied material, tumours were initiated by intracranial implantation of U87MG expressing EGFRvIII, GFP and Luc2. Once tumours were established (as confirmed by bioluminescence imaging) an initial “pre-treatment” biopsy was performed, using GFP expression as a guide. Animals were monitored using the NSS until no evidence of neurological damage was seen. Mice were then treated with dacomitinib, and a second biopsy was performed after two hours. Animals were allowed to recover, and were euthanised once tumour-related morbidity was observed.

**Fig 4:**
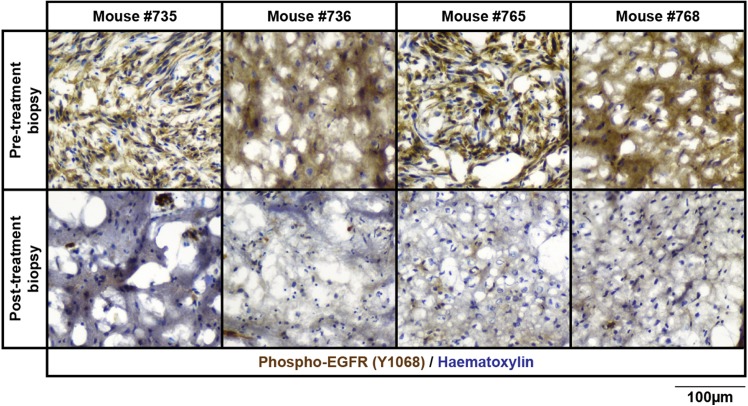
Tumour response to kinase inhibitor therapy can be observed in biopsied tissue. Tumour-bearing animals underwent surgical biopsy (pre-treatment) followed by a period of recovery before each was treated with 30mg/kg dacomitinib to inhibit EGFR. After 2 hours, a second biopsy was performed (post-treatment). Immunohistochemical detection of phosphorylated EGFR (Y1068, brown) in cryosections of biopsied tumours demonstrates that EGFR phosphorylation was significantly reduced after treatment with dacomitinib (lower row).Four representative mice are shown (#735, #736, #765 and #768). Sections were counterstained with haematoxylin (blue). Scale bar represents 100μm.

All biopsied tissue underwent cryo-sectioning and the intensity of immunohistological staining for phospho-EGFR before and after treatment with dacomitinib was compared. Reduced phospho-EGFR was observed in five out of eight of the post-treatment biopsies (Fig 4). Despite minor inconsistencies, these data illustrate that this surgical biopsy technique can isolate tumour tissue which retains biologically relevant information for preclinical assessment of drug treatment.

## Discussion

Preclinical assessment of new therapies for cancer is an essential part of the pharmacological drug development pipeline. This study confirmed that serial brain tumour biopsies can be conducted successfully and reliably in mice to obtain tissue samples for histological processing with minimal impact on neurological morbidity. To evaluate the technique, an orthotopic xenograft model of GBM was used. There are two main reasons for modelling brain tumours in animals. Firstly, to identify genetic and molecular events associated with tumour formation, thereby identifying new targets for treatment and broadening our knowledge of the underlying mechanisms of oncogenesis, and secondly, relevant to this report, to evaluate potential therapeutic strategies [12]. The serial biopsy method described here, has the potential to provide tissue samples at various stages of treatment or tumour development from a single mouse.

Stereotactic needle biopsy is a commonly used procedure to identify lesions in humans that may not be easily accessible surgically or where there is diagnostic uncertainty. It provides useful cellular and histological information about tumour type and grade [2], and has been shown to have a diagnostic accuracy ranging between 80 and 96.7% [13]. Despite being essentially a blind procedure, it is relatively safe with a low reported morbidity of 3.2% and mortality of 0.6% in humans [14]. However, it is associated with incorrect diagnoses or no diagnosis in up to 17% and 35% of cases [2], respectively. Due to the small size of the mouse brain, and the relatively large size of human needle biopsy equipment, this technique is rarely used in murine models. Instead, histological examination after animal sacrifice is more commonly utilised to monitor molecular characteristics within the tumour, requiring the use of large numbers of animals. The process is also time consuming and does not allow the monitoring of temporal changes in the tumour during treatment.

An alternative to needle biopsy is open biopsy: a technique used for human tumours, which allows direct visualisation of abnormal tissues and surrounding structures. This method provides a potential reduction in rates of missed diagnosis experienced with needle biopsy. Moreover, larger tissue samples can be obtained making histological analyses easier, with a lower risk of haemorrhage from blindly probing vessels. However, the procedure may be associated with slightly higher overall risk and may take longer. Here, we observed that the technique of open free-hand biopsy translated better to animal work with mouse models.

GBM in humans is a diffusely infiltrating primary brain tumour. It is known that extent of resection is associated with improved median survival in high grade glioma[15], yet defining a tumour margin at surgery is notoriously difficult. In order to better circumscribe surgical resection 5-aminolevulinic acid (5-ALA), a pro-drug that causes fluorescent protoporphyrins to accumulate in malignant glioma, has been trialled [16, 17]. Using a modified intraoperative microscope the tumour tissue emits red fluorescence under blue light illumination in human patients. In the mouse model described here, GFP, a fluorescent reporter expressed by the tumour cells, was utilised to ensure accurate identification of tumour from surrounding normal brain. In a similar way to 5 ALA, GFP allowed accurate identification of tumour tissue at the time of surgery, and confirmation of tumour in the biopsy sample after removal.

We were able to show a response of the brain tumours to drug treatment as evidenced by a reduction of phosphorylated EGFR in the majority of samples following treatment with dacomitinib. However, this target inhibition was not entirely consistent across all samples. This is likely due to technical reasons, such as inaccurate drug administration. In addition, analysis of the intensity of staining was slightly confounded by the presence of an “edge effect”, or an artefactual increase in staining around the edge of the fixed tissue specimen. This may also have contributed to the variability, which was exacerbated due to the small size of the biopsy. From previous studies, it has been shown that surgical procedures and postsurgical tissue processing can significantly affect gene expression and the EGFR-pathway proteins [18]. As such, it is possible that the variability we experienced with assessment of response to treatment may also be procedurally related. To address this, future studies should aim at standardizing treatment protocols and/or assessing other predictive biomarkers less sensitive to processing artifacts. Despite these sources of error, the biopsy procedure consistently provided quality tissue samples for histological analysis.

Although orthotopic xenografts were used in this study, the results are relevant to other preclinical models of brain cancer. For example, brain tumours in genetically engineered mouse models (GEMMs) can now be identified by small animal MRI. Many spontaneously arising brain tumours in GEMMs are molecularly diverse due to the acquisition of secondary mutations. Mutations affecting the PTEN, TP53 and RB1 pathways are known to be obligate events in the pathogenesis of human glioma. Mouse models that mimic these mutations in the brain develop a range of different astrocytic tumours, including anaplastic astrocytoma (grade III), GBM and gliomatosis cerebri [19]. In addition, these tumours exhibit mutations in multiple different RTKs, such as *Egrf* and *c-Met*. If these tumours were to arise in an easily accessible area, our biopsy method could be advantageous, not only to confirm the type of brain tumour, but also to provide molecular information such that the same mouse could be used for subsequent testing of targeted inhibitors.

To our knowledge, this is the first report of a serial biopsy method for mouse brain tumours. More recently, Kim *et al* designed an optical probe within a 22G needle that could be used to conduct deep brain imaging *in situ* (>1mm). The procedure could be performed in serial experiments relatively rapidly and was shown to be associated with minimal neurological injury [20]. Currently, however, the method is limited to tumour models that express specific fluorescent reporters (such as GFP). As such, the use of the technique is limited, and not able to assess the wide variety of molecular changes within a tumour that can be assessed using a biopsy and histological examination of extracted tissue.

Future directions for this technique include its application in transgenic models and in the analysis of different types of tumours. Anticipated difficulties with the external validation of the technique include problems with locating the tumour for biopsy, and tumours arising in more eloquent areas of mouse brain, such as the cerebellum. However, these difficulties can be overcome with the use of imaging modalities such as MRI to identify the precise location of the tumour and allow determination of stereotactic coordinates prior to biopsy. The analysis of the samples could be extended to include not only IHC but also techniques to genotype the samples, such as DNA copy number analysis to detect amplification in RTK genes. This would not only enable an assessment of response to treatment with novel agents, but also allow evaluation of genetic heterogeneity in serial samples.

## Conclusion

Despite the use of tumour tissue banking, the rarity of brain tumours means that reliable preclinical models are in short supply. The disparity between the low volume of tissue available and the large number of possible therapeutic targets, highlights the importance of tractable mouse models for these diseases. With the trend in the clinic moving towards target-specific chemotherapy, it first needs to be determined which of these agents has potential to be therapeutically beneficial and define the characteristics of tumours that are likely to respond to treatment. The serial biopsy method described here for preclinical brain tumour models will help achieve this goal. The ability to obtain serial tissue samples during the treatment phase will enable more accurate and efficient preclinical assessment of drugs, facilitating a more rapid translation to brain tumour clinical trials.

## Supporting information

S1 Fig Dacomitinib-induced inhibiton of EGFRvIII in mouse brain. Mice bearing orthotopic xenografts of U87MG expressing EGFRvIII (*U87+vIII*) were treated with vehicle or dacomitinib. Tumour tissue was harvested after 2 hours, and inhibition of EGFRvIII was determined by immunohistochemical staining for phosphorylation of tyrosine 1081 (*brown*). Sections were counterstained with hematoxylin (*blue*). Scale bar applies to both images.
(TIF)

S1 Table Minimal data set showing NSS for mice 5 days after implantation (Controls), first biopsy and second biopsy. (DOCX)
