# Correction: A novel technique of serial biopsy in mouse brain tumour models

**DOI:** 10.1371/journal.pone.0214401

**Published:** 2019-03-20

**Authors:** The PLOS ONE Staff

## Notice of Republication

This article was republished on March 13, 2019 to correct for errors in the Data Availability statement introduced during the typesetting process. The publisher apologizes for the errors. Please download this article again to view the correct version.
