# Supplementary figures and images for: A novel technique of serial biopsy in mouse brain tumour models
Source: PLoS One. 2017 Apr 10;12(4):e0175169. doi: 10.1371/journal.pone.0175169 (PMC5386264; doi:10.1371/journal.pone.0175169)

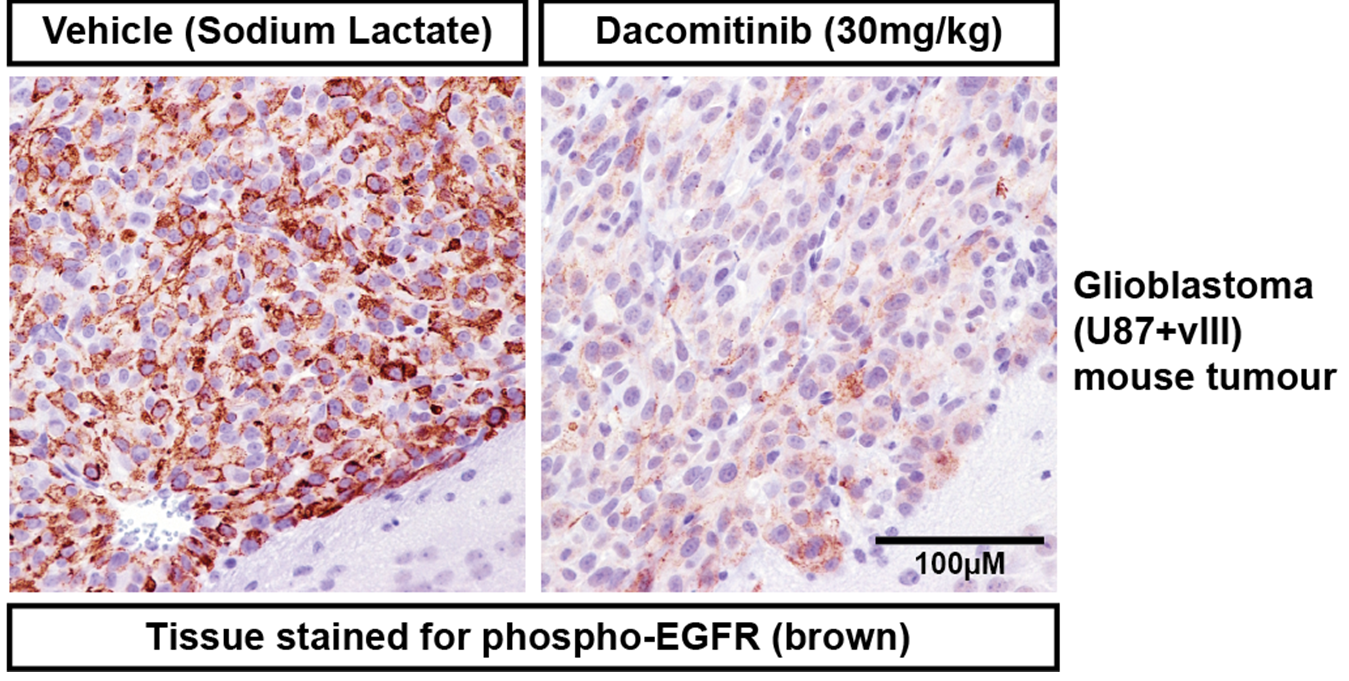

Supplement: S1 Fig — Mice bearing orthotopic xenografts of U87MG expressing EGFRvIII (U87+vIII) were treated with vehicle or dacomitinib. Tumour tissue was harvested after 2 hours, and inhibition of EGFRvIII was determined by immunohistochemical staining for phosphorylation of tyrosine 1081 (brown). Sections were counterstained with hematoxylin (blue). Scale bar applies to both images. (TIF) [file pone.0175169.s001.tif]
