# Supplementary material for: A novel technique of serial biopsy in mouse brain tumour models
Source: PLoS One. 2017 Apr 10;12(4):e0175169. doi: 10.1371/journal.pone.0175169 (PMC5386264; doi:10.1371/journal.pone.0175169)
Supplement: S1 Table — (DOCX) [file pone.0175169.s002.docx]

**Minimum data set of findings from study**

| ***Controls NSS*** |  |  |  |  |  |
| --- | --- | --- | --- | --- | --- |
| **Mouse #** | ***Day 1*** | ***Day 2*** | ***Day 3*** | ***Day 4*** | ***Day 5*** |
| 767 | 1 | 1 | 1 | 0 | 0 |
| 768 | 2 | 0 | 0 | 0 | 1 |
| 770 | 1 | 0 | 0 | 0 | 0 |
| 771 | 1 | 1 | 0 | 0 | 2 |
| 772 | 0 | 0 | 0 | 0 | 1 |
| 738 | 0 | 1 | 1 | 5 | - |
| 739 | 1 | 1 | 0 | 0 | 0 |
| 740 | 1 | 0 | 0 | 0 | 0 |

| ***NSS after 1st biopsy*** | |  |  |  |  |
| --- | --- | --- | --- | --- | --- |
| **Mouse #** | ***Day 1*** | ***Day 2*** | ***Day 3*** | ***Day 4*** | ***Day 5*** |
| 733 | 2 | 1 | - | - | - |
| 735 | 2 | 0 | - | - | - |
| 736 | 3 | 3 | - | - | - |
| 737 | 2 | 2 | - | - | - |
| 763 | 1 | 1 | 2 | 1 | 2 |
| 764 | 2 | 0 | 1 | 2 | 1 |
| 765 | 1 | 1 | 0 | 1 | 2 |
| 766 | 3 | 2 | 2 | 2 | 2 |
| 768 | 3 | 0 | 0 | 1 | 0 |
| 769 | 3 | 3 | 2 | 2 | 2 |
| 770 | 3 | 4 | - | - | - |
| 771 | 2 | 2 | - | - | - |
| 772 | 2 | 1 | 1 | 1 | 1 |
| 826 | 1 | 1 | 1 | 0 | 0 |
| 827 | 0 | 0 | 1 | 1 | 0 |
| 828 | 2 | 2 | 1 | 0 | 0 |
| 829 | 1 | 1 | 0 | 1 | 0 |
| 830 | 0 | 0 | 0 | 0 | 0 |
| 831 | 0 | 0 | 1 | 0 | 0 |
| 832 | 2 | 1 | 1 | 1 | 1 |
| 833 | 1 | 1 | 1 | 1 | 0 |

| ***NSS after 2nd biopsy and PF*** | | |  |  |  |
| --- | --- | --- | --- | --- | --- |
| **Mouse #** | ***Day 1*** | ***Day 2*** | ***Day 3*** | ***Day 4*** | ***Day 5*** |
| 736 | 4 | 3 | 2 | 1 | - |
| 737 | 3 | - | - | - | - |
| 764 | 2 | 1 | 1 | 1 | 1 |
| 765 | 2 | 2 | 2 | 1 | 1 |
| 768 | 3 | 2 | 1 | 1 | 1 |
| 772 | 3 | 3 | 1 | 1 | 1 |
| 826 | 0 | 0 | 1 | 1 | 1 |
| 827 | 1 | 0 | 3 | 0 | 0 |
| 828 | 1 | 2 | 0 | - | - |
| 829 | 0 | 1 | 0 | 1 | 0 |
| 830 | 0 | 1 | 0 | 0 | 0 |
| 831 | 1 | 1 | 2 | 1 | 0 |
| 833 | 0 | 0 | 1 | 2 | 2 |
| 835 | 1 | 1 | 0 | 0 | 2 |

**DOI 10.17605/**[**OSF.IO/CBM7V**](http://osf.io/CBM7V)

**ARK c7605/**[**osf.io/cbm7v**](http://osf.io/cbm7v)
